# Antitumor Effect of the Ethanolic Extract from Seeds of *Euphorbia lathyris* in Colorectal Cancer

**DOI:** 10.3390/nu13020566

**Published:** 2021-02-09

**Authors:** Cristina Mesas, Rosario Martínez, Raúl Ortíz, Milagros Galisteo, María López-Jurado, Laura Cabeza, Gloria Perazzoli, Consolación Melguizo, Jesús M. Porres, Jose Prados

**Affiliations:** 1 Institute of Biopathology and Regenerative Medicine (IBIMER), Center of Biomedical Research (CIBM), University of Granada, 18100 Granada, Spain; cristinam@correo.ugr.es (C.M.); roquesa@ugr.es (R.O.); lautea@ugr.es (L.C.); gperazzoli@ugr.es (G.P.); jcprados@ugr.es (J.P.); 2 Department of Anatomy and Embryology, Faculty of Medicine, University of Granada, 18071 Granada, Spain; 3 Instituto Biosanitario de Granada (ibs. GRANADA), 18014 Granada, Spain; 4 Department of Physiology, Institute of Nutrition and Food Technology (INyTA), Biomedical Research Center (CIBM), Universidad de Granada, 18100 Granada, Spain; rosariomz@ugr.es (R.M.); mlopezj@ugr.es (M.L.-J.); jmporres@ugr.es (J.M.P.); 5 Department of Pharmacology, School of Pharmacy, University of Granada, 18071 Granada, Spain; mgalist@ugr.es

**Keywords:** colon cancer, *Euphorbia lathyris*, ethanolic extract, apoptosis, antiangiogenic effect, cancer stem cells

## Abstract

The seeds of *Euphorbia lathyris* have been used in traditional medicine to treat various medical conditions. However, neither all of their active biocompounds nor the molecular mechanisms underlying their therapeutic effects have been described. A new ethanolic extract of defatted flour from mature seeds of *Euphorbia lathyris* showed a high total polyphenol content and significant antioxidant activity. Chromatographic analysis showed that esculetin, euphorbetin, gaultherin, and kaempferol-3-rutinoside were the most abundant polyphenolic bioactive compounds. Antiproliferative assays showed a high and selective antitumor activity against colon cancer cell lines (T84 and HCT-15). In addition, a significant antiproliferative activity against glioblastoma multiforme cells was also demonstrated. Its mechanism of action to induce cell death was mediated by the overexpression of caspases 9, 3, and 8, and by activation of autophagy. Interestingly, a reduction in the migration capacity of colon cancer cells and a significant antiangiogenic effect on human umbilical vein endothelial cells were also demonstrated. Finally, the extract significantly reduced the subpopulations of cancer stem cells. This extract could be the basis to develop new therapeutic strategies for the treatment of colon cancer, although further experiments will be necessary to determine its in vivo effects.

## 1. Introduction

Population aging, nutrition, and sedentary lifestyle are related to the severe increase in the prevalence of colorectal cancer (CRC), which currently represents the third most common cancer worldwide [1,2]. Surgical treatment is used in patients with non-metastatic CRC [3], but chemotherapy is still required in advanced (metastatic) cancer. Although monoclonal antibodies and multiple kinase inhibitors have enhanced its prognosis [4], the success of chemotherapy against this tumor is yet subject to the discovery of new anticancer agents. In this context, the activity of plant extracts or derivatives on the viability and survival of tumor cells has gained increasing interest [5].

Euphorbia, a therapeutic resource of traditional Chinese medicine, has a great potential based on its known anticancer properties. Euphorbia comprises a great diversity of plants from the spurge family (Euphorbiaceae), containing more than 2000 species [6]. The Euphorbiaceae family comprises a great chemical variety that includes a wide range of diterpenoids with therapeutic activities such as antiproliferative, anti-inflammatory, antiviral, and immunomodulatory properties [7,8,9,10,11]. In fact, some diterpenoids from *Euphorbia esula* L. showed antimalarial [12], pro-inflammatory, and antitumor activities against multidrug-resistant (MDR) and non-resistant human gastric cancer cells [13,14]. Ingenol mebutate (Picato^®^, Leo Pharma Laboratories, Barcelona, Spain) from *Euphorbia peplus* L. is a diterpene used for the topical treatment of actinic keratosis and skin cancer [6]. Euphol, a tetracyclic triterpene alcohol present in the sap of Euphorbia and in the latex of some Euphorbia species [15], showed anti-inflammatory, contraceptive, and even antitumor properties [16]. Recently, an extract of *Euphorbia esula* L. showed antiproliferative activity against lung, cervical, gastric, breast, and liver cancers [17], while diterpenoids isolated from an ethanolic extract of *Euphorbia helioscopia* showed cytotoxicity in renal cancer cell lines [18]. Interestingly, most of the studies conducted on Euphorbia focused on the presence of biologically active natural products in latex [19,20]. For example, analysis of the latex of *Euphorbia umbellata* revealed its ability to modulate functions of the immune system, contributing to the activity of the complement pathways [21]. Similarly, the latex from Euphorbia tirucalli showed an efficient decrease in tumor growth in rats [22].

Within the Euphorbiaceae family, the seeds of *Euphorbia lathyris* have been used in traditional Chinese medicine [19] for hydropsy, ascites, constipation, amenorrhea, terminal schistosomiasis, and snakebites [23,24]. These seeds contain numerous diterpenes that showed antitumor activity against cell lines with and without resistance by MDR-mediated drugs [25,26]. Bicchi et al. [27] described the presence of diterpenoid fractions corresponding to ingenol and Euphorbia factors in seed oil from *Euphorbia lathyris*, whereas Jiao et al. [28] analyzed an ethanolic extract from seeds of *Euphorbia lathyris* and identified 22 types of compounds, including diterpenoids, triterpenoids, steroids, fatty acid esters, and coumarins. Moreover, Teng et al. [29] obtained an ethanolic extract from seeds of *Euphorbia lathyris* using 95% ethanol under reflux and reported the existence of lathyran-type diterpenoids, highlighting five diterpenes with a lathyran structure (the Euphorbia factors L1, L2, L3, L8, and L9). These compounds showed cytotoxic activity against lung, nasopharyngeal, and breast (triple negative) carcinomas. On the other hand, Duan et al. [30] demonstrated the activity of Euphorbia seeds against leukemia and skin cancer. A recent study by Fan et al. [31] using the L2 factor from Euphorbia seeds showed specific antiproliferative activity against hepatocellular carcinoma. Finally, the modulating effects of MDR and P-glycoprotein have also been studied using extracts of *Euphorbia lathyris* [32].

Despite the current knowledge about chemical compounds of *Euphorbia lathyris* seed, it is still necessary to explore new pharmacological activities as well as their mechanisms of action [33]. The aim of this study was to obtain a functional extract from seeds of *Euphorbia lathyris* with antitumor activity against CRC, as well as to elucidate the molecular mechanisms by which the extract induces cell death. Our results demonstrated a surprisingly high antiproliferative activity of the ethanolic extract of *Euphorbia lathyris* against human colon cancer cells, which should be further analyzed in order to obtain new molecules that enhance the currently available therapies against this type of tumor.

## 2. Materials and Methods

### 2.1. Plant Material

Mature seeds of *Euphorbia lathyris* (S3201 variety) were provided by Agrointec Solution S.L, which developed a seed defatting process that separates the oleaginous part of the mature seed by means of a cold seed oil extraction press (KOMET, IBG Monforts, GER). Defatting did not exceed a temperature of 40 °C and was characterized by an average working speed of 2–3 kg seed/h and oil to seed conversion yield ranging from 15 to 25%. As a result of this process, defatted flour with a particle size between 100 µm and 150 µm was obtained and stored in absence of light and at −20 °C until use.

### 2.2. Ethanolic Extract

**Figure 1:**
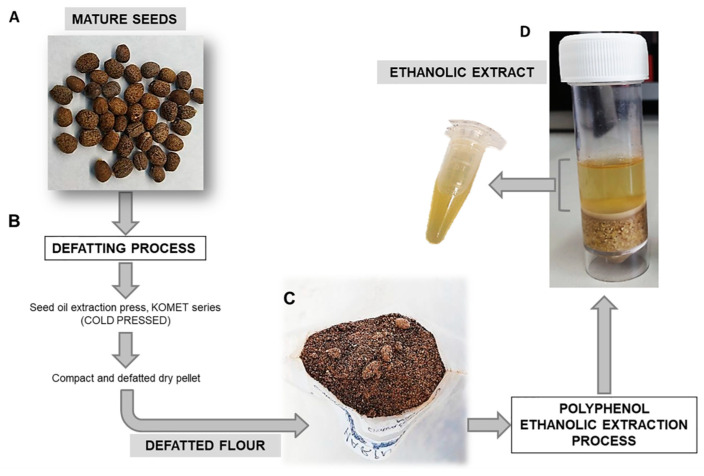
Representative image of ethanolic extracts obtained from *Euphorbia lathyris*. (**A**) *Euphorbia lathyris* mature seeds; (**B**) Schematic representation of the defatting process from mature seeds; (**C**) Macroscopic image of defatted flour from mature seeds; (**D**) Macroscopic image of the polyphenol ethanolic extraction process and the final extract obtained.

Defatted flour from mature seed of *Euphorbia lathyris* were used to obtain the ethanolic extract rich in polyphenols. The ethanolic extract process was developed mixing 5 g of defatted flour with 15 mL of hydroalcoholic solution (Ethanol: type I water: 12 N HCl; 50:50:0.2) at pH 2 and 4 °C in a reduing atmosphere (with nitrogen) for 30 min in a magnetic stirrer. After 30 min stirring, the extract was centrifuged at 3000 rpm for 5 min. The supernatant was stored, and the pellet was recovered to repeat the process. Finally, all the supernatants (ethanolic extract) were mixed and stored at −20 °C (Figure 1). The whole process was done in triplicate. To determine the ethanolic extract yield and extract concentration, aliquots (1 mL) were treated to evaporate ethanol using a vacuum evaporator (Savant DNA120 SpeedVac Concentrator, ThermoSci, Waltham, MA, USA) (Eppendorf Concentrator 5301). The evaporated extracts were frozen in liquid nitrogen and freeze-dried (Cryodos-50 lyophilizer, TELSTAR, Madrid, Spain) during 24 h. Then, the dry weight of the extract was calculated by subtracting the weight of the empty recipient containing each aliquot and referred to a volume of 1 mL of initial extract, to the total volume of extract obtained, and, finally, to the grams of flour.

### 2.3. Assessment of Total Polyphenol Content

Total polyphenol content was determined in ethanolic extracts from the defatted flour of *E. lathyris* using the Folin–Ciocalteu method according to a previous report [34]. Different concentrations of gallic acid (between 0 and 500 μg/mL) were used as calibration curve samples. The results were expressed as gallic acid (GA) equivalents (μg gallic acid*mg^−1^ of ethanolic extract).

### 2.4. Antioxidant Activity

The reducing capacity of Fe^3+^ to Fe^2+^ by the different extracts was measured spectrophotometrically by the technique of Duh et al. [35] as described by Kapravelou et al. [34]. A standard curve of GA concentrations ranging from 0 to 80 μg/mL was used in the analysis.

### 2.5. Chromatographic Studies

Bioactive compounds present in ethanolic extracts from the defatted flour of E. lathyris were analyzed by Ultra Performance Liquid Chromatography (UPLC) coupled with a Quadrupole Time of Flight (QTOF) Mass Spectrometer (Synap G2, Waters, Milford, MA, USA). For that purpose, polyphenols were separated analytically by an Acquity HSS T33 analytical column (100 mm × 2.1 mm internal diameter, 1.8 μm; Waters, Milford, MA, USA). The mobile phase of the column consisted of a gradient formed by combining solvent A (deionized water with 0.5% acetic acid) and solvent B (acetonitrile with 0.5% acetic acid). The flow rate of the mobile phase was 0.4 mL/min. High-resolution mass spectrometry analysis was carried out in negative electro spray ionization (ESI-ve) and spectra recorded over a 50–1200 mass/charge (*m/z*) range.

The chromatograms obtained were manually analyzed using the MassLynx V4.1 software. The presence of the compounds was validated by matching at least 3 sub-fragments obtained from the Chemspider database. The presence of specific compounds was corroborated and quantified using the following standards: esculetin (68923, Sigma-Aldrich, Madrid, Spain), euphorbetin (35897-99-5, Cymit Química, S.L. Barcelona, Spain), gaultherin (490-67-5, Cymit Química, S.L. Barcelona, Spain), and kaempferol-3-rutinóside (17650-84-9, Cymit Química, S.L. Barcelona, Spain).

### 2.6. Cell Culture

The T84 (sensitive to chemotherapy) and HCT15 (resistant to chemotherapy) human colon adenocarcinoma cell lines were purchased from the American Type Culture Collection (Rockville, MD, USA). The non-tumor colon cell line CCD18 (human colon epithelial cell line) was used as control. Human glioblastoma (SF268, SK-N-SH, A172, LN229), human pancreatic adenocarcinoma (PANC-1) and human breast cancer (MCF-7) cell lines were provided by the Scientific Instrumentation Center (CIC, Granada University, Granada, Spain). Both SF268 and SK-N-SH cells showed resistance to temozolomide (TMZ) through O-6-Methylguanine-DNA Methyltransferase (MGMT) expression. All cell lines were grown in Dulbecco’s Modified Eagle’s Medium (DMEM) (Sigma-Aldrich, Madrid, Spain) supplemented with 10% heat-inactivated fetal bovine serum (FBS) (Gibco, Madrid, Spain) and antibiotics (gentamicin/amphotericin-B + penicillin/streptomycin) (Sigma Aldrich, Madrid, Spain) at 1% and maintained in an incubator at 37 °C and 5% CO_2_ humidified atmosphere.

### 2.7. Cell Viability Assay

Cells were seeded in 48-well plates with DMEM (300 μL) at a density of 4 × 10^3^ cells/well in CCD18 and MCF-7, 5 × 10^3^ cells/well in T84, HCT15, SF-268, A-172, and SK-NSH cell lines, and 8 × 10^3^ cells/well in PANC-1 and LN-226 cell lines, respectively. After 24 h, cell cultures were exposed to the ethanolic extract, which was previously evaporated to avoid ethanol toxicity. The extract was easily dissolved in DMEM without any additional solvent, and no signs of contamination were observed along the experiment due to sterile nature of an ethanolic extract and the cell growth in culture medium treated with antibiotics. Then, cell cultures were exposed to increasing concentrations of the evaporated ethanolic extract of defatted flour for 72 h. After the incubation time, cells were fixed with 10% trichloroacetic acid (TCA) for 20 min at 4 °C. Once dried, the plates were stained with 0.4% sulforhodamine B (SRB) in 1% acetic acid (20 min, in agitation). After three washes with 1% acetic acid, SRB was solubilized with Trizma^®^ (10 mM, pH 10.5). Finally, the optical density (OD) at 492 nm was measured in a spectrophotometer EX-Thermo Multiskan. Cell survival (%) was calculated according to the following equation:

$$\mathrm{Cell}\;\mathrm{survival}\;\left(\%\right)=\;\frac{\mathrm{Treated}\;\mathrm{cells}\;\mathrm{OD}-\mathrm{blank}}{\mathrm{Control}\;\mathrm{OD}-\mathrm{blank}\;}\times \;100.$$

In addition, half maximal Inhibitory Concentration(IC50) was calculated (GraphPad Prism 6 Software, La Jolla, CA, USA).

### 2.8. Western Blot Analysis

Western blot analysis using proteins obtained from T84 cells exposed to the ethanolic extract obtained from the defatted flour (IC50 and twice IC50) during 12 h and 24 h was performed. First, cells were collected, centrifuged, and total proteins were extracted with RIPA (Radio-Immunoprecipitation Assay) lysis buffer (Thermo Fisher Scientific, Waltham, MA, USA) to determine protein concentration using Bradford. For electrophoresis, 40 µg protein of each sample were heated at 95 °C for 5 min and separated in 10% SDS-PAGE gel in a Mini Protean II cell (Bio-Rad, Hercules, CA, USA). Proteins were transferred to a nitrocellulose membrane with a 45 µm pore size (200 V at room temperature (RT) for 1 h) (Millipore, Burlington, MA, USA) and treated with blocking solution (Phosphate-Buffered Saline (PBS)-0.1% Tween-20 + 5% (*w/v*) milk powder) for 1 h. After washing three times with PBS-0.1% Tween-20, membranes were incubated with the primary antibody overnight at 4 °C (rabbit polyclonal Immunoglobulin G (IgG) anti-caspase-3 (sc-271759), 1:500 dilution; anti-caspase-8 (sc-166320), 1:1000 dilution; and anti-caspase-9 (sc-133109), 1:1000 dilution) (Santa Cruz Biotechnology, Santa Cruz, CA, USA). After three washes, the membranes were incubated for 1 h at RT with the secondary antibody peroxidase conjugate (1:5000 dilution) (Goat anti-mouse IgG-HRP, Santa Cruz Biothecnology, CA, USA). In addition, anti-β-actin IgG (A3854, Sigma Aldrich, Madrid, Spain) (1:10,000 dilution) was used as an internal control. Signals were detected by an ECLTM Western blot detection reagent (Enhanced Chemiluminescence; Bonnus, Amersham, Little Chalfont, UK) [36]. Once the Western blot was performed, the bands obtained in the gels were analyzed using Quantity One analytical software (Bio-Rad, Hercules, CA, USA).

### 2.9. Wound-Healing Assay

To determine the tumor cell migration capacity of cell lines and, therefore, their invasiveness and ability to generate metastases, an in vitro migration assay was performed. T-84 cells were seeded in 12-well plates and grown to 100% confluence in standard culture conditions. Once confluence was reached, a “wound” was manually performed with a sterile tip following Grada et al. [37], and the medium was substituted for serum-free DMEM. Immediately, cells were exposed to the ethanolic extract obtained from defatted mature seeds (non-cytotoxic dose, IC5) during 72 h. Images were obtained at different times (0, 8, 24, 48, and 72 h) to observe cell migration in comparison to the control (cells without treatment). To evaluate the effect of the ethanolic extract, the percentage of migration was calculated by measuring the area free of tumor cells at different times (Image J software) (https://imagej.nih.gov/ (accessed on 15 October 2019)).

### 2.10. Cell Cycle Analysis

The cells were seeded in 6-well plates at a density of 30 × 10^4^ cells/well. After 24 h, the culture medium was removed, and a serum-free culture medium was added to arrest the cell cycle. Then, the culture medium was replaced by DMEM with serum, including extract concentrations equivalent to the IC25 and IC50. After 48 h of incubation, the trypsinized cells were fixed with 70% ethanol in agitation at 4 °C (1 h) and washed twice with PBS. Finally, cells were processed using the PI/RNASE Solution Kit (Immunostep, Salamanca, Spain) to quantify the total content of cellular DNA by FACScan flow cytometer (Becton Dickinson, San Jose, CA, USA) using FlowJo software (Treestar, Ashland, OR, USA), determining the phase of the predominant cell cycle.

### 2.11. Alpha-Tubulin Immunofluorescence Assay

T-84 cells were seeded in an 8-well chamber at a density of 20 × 10^3^ cells/well. Then, cells were exposed to the ethanolic extract (IC25 and IC50) from the defatted flour of E. lathyris. After 24 h, the cells were fixed with 4% formaldehyde in PBS-0.1% Tween for 25 min at RT, permeabilized with 0.1% Triton X-100, and blocked with a solution of goat serum for 60 min. Then, the cells were incubated with the primary anti-α-tubulin antibody 1:250 (*v/v*) (Sigma Aldrich, Madrid, Spain) for 1 h at RT, washed (three times) with PBS-0.1% Tween, and incubated with the secondary antibody (Alexa-Fluor 488-conjugated secondary antibody; Cell Signaling Technologies, Spain; 1:200 dilution) for 60 min at RT in darkness. Hoechst dye (1:2000) was used to stain nuclei. Cells were observed by fluorescence microscopy (Leica Microsystems, Wetzlar, Germany).

### 2.12. Angiogenesis Assays

Angiogenesis assays were carried out using human umbilical vein endothelial cells (HUVECs), which were purchased from the American Type Culture Collection (Rockville, MD, USA). HUVECs were cultured in 0.1% gelatin-coated flasks in Endothelian Cell Growth Medium 2 (EGM-2) (Lonza Bioscence, Barcelona, Spain) supplemented with EGMTM-2 Endothelial SingleQuotesTM Kit (Lonza Bioscence) including FBS, human Epidermal Growth Factor (hEGF), Vascular Endothelial Growth Factor (VEGF), R3-Insulin-like Growth Factor-1 (R3-IGF-1), ascorbic acid, hydrocortisone, human Fibroblast Growth Factor-Beta (hFGF-β), heparin, and antibiotics (Sigma Aldrich, Madrid, Spain) at 37 °C in 5% CO_2_ humidified atmosphere. Conditioned medium of the T-84 culture cells treated with the ethanolic extract from the defatted flour at IC25 and IC50 concentrations during 24 h was obtained. After 24 h incubation, the medium was removed and replaced by new medium (without the ethanolic extract). Then, HUVECs were seeded in 96-well plates (50 × 10^3^ cells/well) previously coated with 50 µL of matrigel (Corning, New York, NY, USA) and were grown (12 h) in a medium without FBS. HUVECs were exposed to the conditioned medium. Calcein was added to the cells, and the formation of tubes was visualized under optical and fluorescence microscopy at 3 h and 7 h. The images were analyzed using ImageJ software to evaluate the degree of microvascular network sprouting.

### 2.13. Real Time PCR

To determine the antitumor activity of the ethanolic extract in colon cancer stem cells, T84 cultured cells were exposed to ethanolic extract (IC50) of defatted seed flour from *Euphorbia lathyris*. After 72 h, the medium was removed, and cells were washed with PBS and then cultured. Total RNA was extracted using Trizol Reagent (RNeasy Mini Kit, Qiagen, MD, USA), quantified with NanoDrop 2000 (Thermo Fisher, Waltham, MA, USA), and converted (1 µg of RNA) into cDNA using a retro-transcriptase kit (Promega, Madison, WI, USA) following the manufacturer’s instructions. Real-Time PCR was carried out using SYBR green supermix (Taq Universal SYBR Green Supermix) (Bio-Rad Laboratories, Hercules, CA, USA). The quantitative RT-PCR primers and annealing temperatures (Tm) used are listed in Supplementary Materials Table S1. The analyzed genes were CD24, CD44, SOX2, OCT4, and NANOG. Gene expression data were normalized with Glyceraldehyde-3-phosphate dehydrogenase (GAPDH). All quantitative RT-PCR assays were performed in an ABI 7900 system (ABI), and the 2−∆∆Ct method was applied to calculate relative expression levels.

### 2.14. Lysotracker Labeling

To determine the presence of autophagy vesicles, LysoTracker^®^ Red DND-99 (Thermo Fisher Scientific, Waltham, MA, USA), a fluorescent red dye used for labeling and monitoring of acidic organelles was used. T-84 cells were exposed to the ethanolic extract (IC50) during 24 h, washed in PBS, and loaded with Lysotracker 50 nM for 30 min at 37 °C. Cells were washed again with PBS and stained with DAPI (1:1000). Finally, cells were observed under fluorescence microscopy.

### 2.15. Statistic Studies

All of the analyses were carried out by triplicate, and the results are presented as means ± standard deviation (SD). Statistical analysis was performed using Student’s *t*-tests with the Statistical Package for the Social Sciences (SPSS) v.20 software. Data with *p* < 0.05 were considered as statistically significant.

## 3. Results

### 3.1. Analysis of Yield and Antioxidant Activity

The ethanolic extract from the defatted flour of mature seeds of *Euphorbia lathyris* (variety S3201) showed a high extraction yield (117.5 ± 9.22 mg/g flour). In addition, the extracts exhibited high total polyphenol content (33.5 ± 5.79 μg GA equivalents/mg extract) and demonstrated significant antioxidant activity assessed by the Fe-reducing capacity (23.0 ± 1.92 μg GA equivalents/mg extract) (Table 1).

### 3.2. Mass Spectrometry Analysis of Bioactive Compounds from the Ethanolic Extract of Euphorbia Lathyris

**Figure 2:**
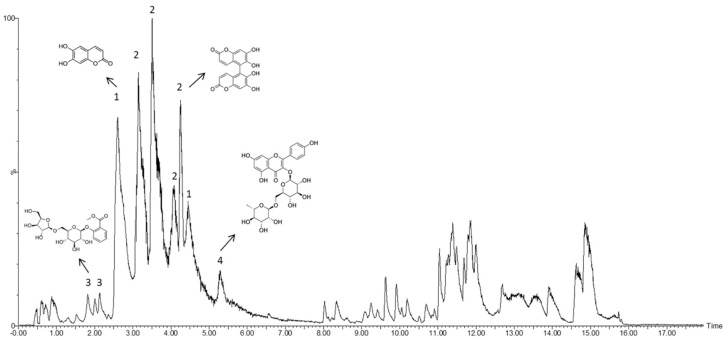
High-performance liquid chromatography chromatogram of polyphenols obtained from the ethanolic extract of *Euphorbia lathyris*. Peak assignment: 1: Esculetin, 2: Euphorbetin, 3: Gaultherin, 4: Kaempferol-3-rutinoside.

To determine bioactive compounds present in the ethanolic extract from the defatted flour of E. lathyris, a chromatographic analysis was carried out (Figure 2).

Analysis of the chromatogram peaks showed a relevant group of bioactive compounds, namely esculetin, euphorbetin, gaultherin, and kaempferol-3-rutinoside (Table 2). To confirm and quantify the most relevant bioactive compounds in the extract, we used isolated standards. Accordingly, the concentration of esculetin, euphorbetin, and kaempferol-3-rutinoside was measured. As shown in Table 3, the biocompound with a highest concentration in the ethanolic extract from the defatted flour of E. lathyris was esculetin (267.68 ± 34.16 ppm).

### 3.3. Effect of the Ethanolic Extract of Euphorbia Lathyris on Cell Viability

The ethanolic extract from the defatted flour of *Euphorbia lathyris* was assayed in colon cancer cells with different degrees of chemo-resistance to determine its antitumor activity. As shown in Table 4, the extract showed a lower IC50 in the T-84 cancer cell line (16.3 ± 2.54 µg/mL) compared to the chemo-resistant colon cancer cell line HCT-15 (IC50 of 72.9 ± 1.27 µg/mL). In contrast, its antiproliferative effect on the normal colon cell line CCD18 was low (266.0 ± 18.5 µg/mL) compared to tumor cells. As positive control, 5-fluoroacile (5-Fu) was tested. Based on these results, the ethanolic extract was assayed in glioblastoma multiforme, pancreatic adenocarcinoma, and breast cancer cell lines. As shown in Table 5, the ethanolic extract from the defatted flour of *Euphorbia lathyris* exhibited antitumor activity in the glioblastoma multiforme cell line, with a lower IC50 in A-172 cells (18.6 ± 1.64 µg/mL) compared to the other cell lines (LN-229, SF-268, and SK-N-SK). With regard to pancreatic adenocarcinoma and breast cancer cell lines, IC50 values were higher than in the other tumor cell lines (185.8 ± 25.8 µg/mL and 89.6 ± 6.29 µg/mL, respectively).

### 3.4. Effect of the Ethanolic Extract of Euphorbia Lathyris on the Cell Cycle

**Figure 3:**
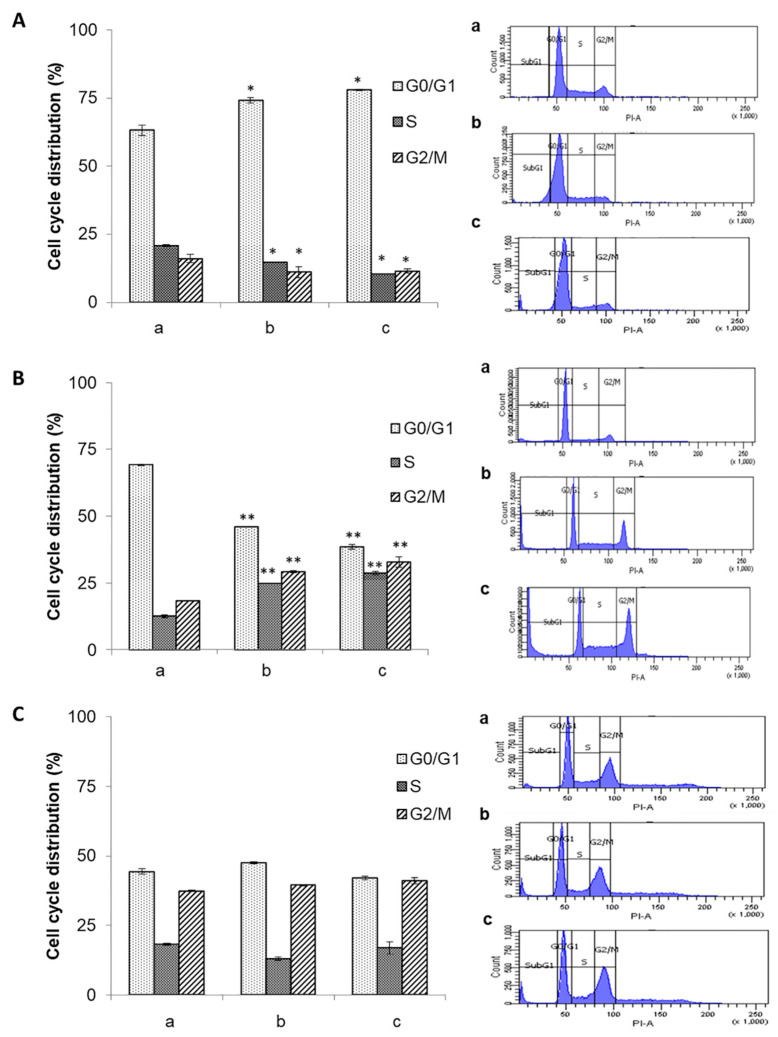
Modulation of the cell cycle by the ethanolic extract from defatted seeds of *Euphorbia lathyris*. Cell lines were treated with IC25 and IC50 doses of the extract. (**A**) T84 cell line; (**B**) HCT-15 cell line; and (**C**) CCD18 cell line (cycle (a: control; b: IC25; c: IC50). Results of the FACScan analysis are expressed as the percentage of labeled cells in each cell cycle phase. Data are presented as mean ± standard deviation of triplicate cultures. * Data with significant differences between treated and non-treated cells (*p* < 0.05). ** *p* < 0.01, vs. the respective control group.

To determine the possible mechanism of action of the ethanolic extract from the defatted flour of *Euphorbia lathyris*, cell cycle studies were conducted in T84, HCT-15, and CCD18 cells treated with the extract. Treated HCT-15 cells showed a dose-dependent increase in G2/M and S phases. Conversely, G0/G1 phase accumulation was observed in T84 tumor cells (Figure 3A,B). Interestingly, no modifications in cell cycle were observed in CCD18 non-tumor cells after exposure to the ethanolic extract (Figure 3C).

### 3.5. Analysis of Cell Migration

**Figure 4:**
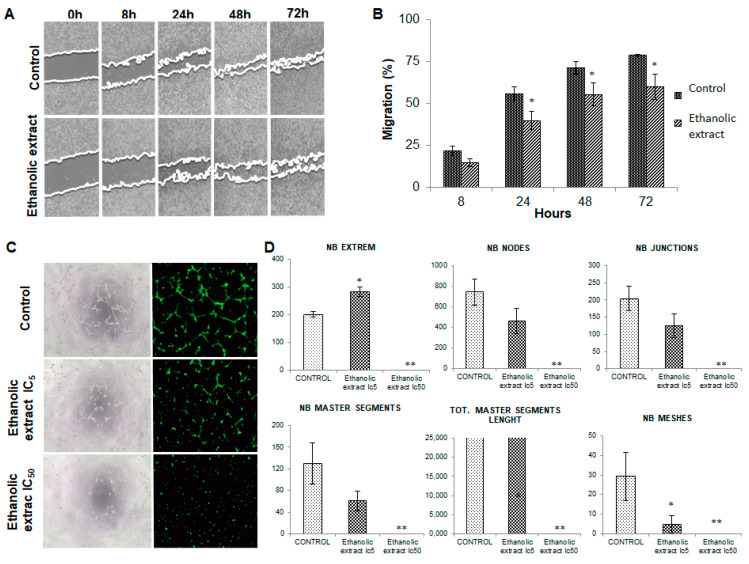
Analysis of migratory capacity and antiangiogenic effect. (**A**) Representative images from T84 colon tumor cell migration at different times (0, 8, 24, 48, and 72 h) after exposure to a subcytotoxic dose (Ic5) of the ethanolic extract of defatted flour from mature seeds of *Euphorbia lathyris*. (**B**) Graphic representation of the percentage of T84 colon tumor cell migration. Data are presented as mean ± standard deviation of triplicate cultures in each column. * Data with significant differences between treated and non-treated cells (*p* < 0.05). (**C**) Representative optical microscopy images of blood vessel formation by human umbilical vein endothelial cells (HUVECs) after exposure to conditioned media obtained from T84 colon cancer cells treated with the ethanolic extract (IC_5_ and IC50). Fluorescence microscopy images of blood vessels formation by HUVECs after exposure to the conditioned medium. (**D**) Graphical representation of different angiogenic parameters measured in fluorescence microscopy images, including number of extreme (NB extreme), formation of nodes (NB nodes), number of master segments (NB master segments), total master segments segments length (TOT. master segments length) and number of junctions (NB junctions). EE, ethanolic extracts. Data are presented as mean ± standard deviation of three independent experiments; * *p* < 0.05 vs. the respective control group, ** *p* < 0.01 vs. the respective control group.

To assess the effects of the ethanolic extract on tumor cell migration capacity, the migration characteristics of T84 cultured cells were analyzed by a cell wound-healing assay. Results showed that non-cytotoxic doses of the extract (IC5) induce a decrease in tumor cell migration vs. control (non-treated) cells. In fact, the modulation of migration could be detected as early as 8 h after wound induction. However, the most significant changes were detected at the end of the experiment (72 h), when an 18.68% decrease in migration was observed in cells exposed to the extract compared to the control cells (Figure 4A,B).

### 3.6. Modulation of Angiogenesis by the Ethanolic Extract of Euphorbia Lathyris

To determine whether our ethanolic extract of *Euphorbia lathyris* was able to modulate angiogenesis via interaction with endothelial cells, HUVEC cultures were exposed to conditioned media obtained from T84 cell cultures treated and non-treated with the extract. As shown in Figure 4C, conditioned media significantly inhibited the formation of blood vessels by HUVECs relative to the control group (HUVECs without exposure). The quantification of vessel formation (number of extremities, nodes, junctions, meshes, master segments formed, and length of master segments) showed a significant reduction compared to the control cells (Figure 4D).

### 3.7. Analysis of Alteration of Stemness Markers

**Figure 5:**
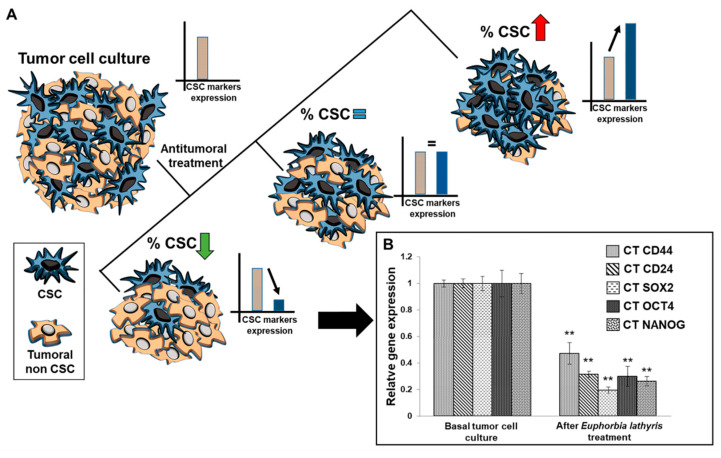
RT-qPCR analysis of cancer stem cell markers. (**A**) Scheme of the possible responses after antitumor treatment in the basal population of tumor cells. The expression levels of cancer stem cell (CSC) markers are related to the percentage of CSC in the tumor culture after antitumor treatment. (**B**) Representative graph showing the relative gene expression of stemness-related markers before (basal) and after exposure to the ethanolic extract from the defatted flour of *Euphorbia lathyris*. Data are presented as mean ± standard deviation of three independent experiments; ** *p* < 0.01 vs. the respective control group.

An RT-qPCR analysis was performed to determine the modulation of stemness-related marker expression in T84 colon cancer cells after exposure to the ethanolic extract from the defatted flour of seeds of *Euphorbia lathyris*. Results demonstrate that the extract, at IC50 doses, was able to significantly decrease the expression of CD44, CD24, NANOG, SOX-2, and OCT-4, indicating a reduction in the amount of cancer stem cells (CSCs) in the culture (Figure 5).

### 3.8. Molecular Analysis of Cell Death Induction by the Ethanolic Extract of Euphorbia lathyris

**Figure 6:**
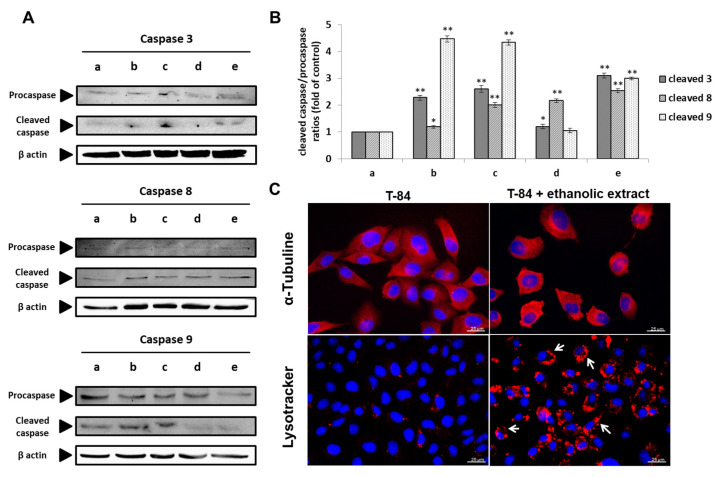
Mechanism of cell death by the ethanolic extract of *Euphorbia lathyris*. (**A**) Western blot analysis of expression of procaspase and cleaved caspase 3, 8, and 9 in T84 cells before treatment (a) and after exposure to the ethanolic extract during 12 h with IC50 dose (b), 12 h with twice IC50 dose (c), 24 h of treatment with IC50 dose (d), and 24 h with twice IC50 dose (e). (**B**) Graphic representation of Western blot densitometry analysis of bands corresponding to coefficient between cleaved caspase and procaspase 3, 8, and 9 expression after treatment with the ethanolic extract (IC50 dose and twice IC50 dose) at different time (12 h and 24 h) (b–e) compared to the untreated control (a). Data are presented as mean ± standard deviation of three independent experiments; * *p* < 0.05 vs. control group; ** *p* < 0.01 vs. control group. (**C**) Representative microscopy images of fluorescence emission from lysosomal and α-tubulin probing of T84 cell line exposed to the ethanolic extract. Formation of autophagic vesicles was evident in the treated cells (arrows).

In order to determine the molecular mechanism of cell death induced by the ethanolic extracts from the defatted flour of seeds of *Euphorbia lathyris*, caspase expression, cell microtubules polymerization/depolymerization, and autophagy were analyzed. As shown in Figure 6A,B, the ethanolic extracts induced T84 cell apoptosis mediated by the caspase pathway, resulting in a significant decrease in the expression of procaspase 9, 8, and 3 and a significant increase in the expression of cleaved caspase 9, 8, and 3 compared to control cells. In addition, the significant formation of autophagy vesicles around the cell nucleus was detected in cell lines exposed to the extract (Figure 6C). Conversely, as shown in Figure 6C, immunofluorescence analysis using α-tubulin did not demonstrate significant differences in microtubule polymerization/depolymerization between T84 cells exposed and non-exposed to the ethanolic extract

## 4. Discussion

Despite therapeutic advances in cancer chemotherapy, current treatments of this disease are far from satisfactory. Treatment failure is related to the known low selectivity of drugs against tumor cells, their side effects, and the generation of drug resistance mediated by several mechanisms. Therefore, novel approaches are required to develop therapeutic alternatives that can improve cancer treatment or enhance the antitumor effects of existing chemotherapeutic agents [38,39]. In this context, numerous natural chemotherapeutic or chemopreventive agents have been isolated and are currently being used for cancer treatment.

Euphorbia species such as *Euphorbia lathyris* have proved to be an excellent resource for obtaining agents with antitumor effects [24]. Most studies have focused on the accumulation of diterpenes in the seeds of Euphorbia, namely ingenol factors with antiproliferative activity. Bicchi et al. [27] described these diterpenoid fractions in the oil obtained from *Euphorbia lathyris* seeds. Furthermore, Euphorbia roots also contain diterpenoids with biological activity [8,40,41]. In fact, the roots of *Euphorbia lathyris* have been recently described as a new source of Euphorbia biomolecules that may show additional benefits [42]. We applied a defatting process to the mature seeds of *Euphorbia lathyris* (variety S3201), not only to increase the concentration of polyphenols with high bioactive capacity in the seed flour, but also to avoid interferences in the extraction process caused by the high fat content of Euphorbia seeds, thus ensuring a high extraction yield. In addition, our ethanolic extraction methodology aimed to avoid potential toxicity from currently used organic extraction solvents to obtain an extract with potential clinical application, since ethanol is used regularly and at low concentrations for the administration of active ingredients. In fact, as a result of our extraction process, ethanolic extracts from the defatted flour showed both high total polyphenol content (33.5 ± 5.79 μg GA equivalents/g flour) and antioxidant activity (23.0 ± 1.92 μg GA equivalents/mg extract). Other extraction methods used in *Euphorbia lathyris* invalidated any possible clinical application. For instance, Zhang et al. [43] used acetone and ethyl acetate, a highly toxic compound for cells, to determine the antioxidant capacity of the extract, but not its antitumor capacity. In addition, Nam and Lee [44] used methanol, which exhibits great toxicity in vivo. Finally, extraction processes by means of a petroleum ether partition (after obtaining a 95% ethanol extract at reflux) showed enrichment of terpenes in the Euphorbia extract, but its potential use in patients seems unlikely [29,30] unless further treatment to eliminate the organic solvents would be applied. On the other hand, although some authors also used ethanol extraction from *Euphorbia lathyris* seeds, their methodologies are significantly different than ours in terms of ethanol concentration, pH, temperature, or extraction process. For instance, Teng et al. [29] used 95% ethanol, obtaining a variety of compounds in the extract that significantly differ from those obtained with our methodology. In fact, these authors mainly obtained diterpenoids from the Latin-type seed (Euphorbia factors L1, L2, L3, L8, and L9), which were not present in our extract. These compounds showed some cytotoxicity only against breast and lung cancer cell lines. Recently, Fan et al. [31] demonstrated that the L2 factor from Euphorbia seeds showed antiproliferative activity against a very specific type of tumor, namely hepatocellular carcinoma. In contrast, our ethanolic extract was especially rich in phenolic compounds (33.52 ± 5.79 µg GA equivalents/mg extract), which led us to conduct a more in-depth analysis of its composition.

Chromatographic analysis of the ethanolic extract from the defatted flour of *Euphorbia lathyris* showed the presence of esculetin, euphorbetin, gaultherin, and kaempferol-3-rutinoside, which was corroborated by the use of certified standards. Interestingly, some of these compounds have been reported to exhibit antiproliferative activity, but they were analyzed independently and obtained from specialized vendors rather than from *Euphorbia lathyris* extracts. For example, Lee et al. [40], Wang et al. [45], Park et al. [46], and Turkekul et al. [47] demonstrated the antitumor effect of esculetin, a coumarin derivative, against non-small cell lung cancer (NCI-H358 and NCI-H1299), leukemia cells (HL-60), colon cancer cell lines such as HCT116, and prostate cancer cells (PC3, DU145, and LNCaP). However, as far as we are concerned, this is the first antitumor activity assay of the complete ethanolic extract from the defatted flour of *Euphorbia lathyris*. Not only did this extract show a potent antitumor activity in non-resistant T-84 colon cancer cells (IC50: 16.29 µg/mL) but also in chemo-resistant colon cancer cells such as the HCT-15 cell line (IC50: 72.9 µg/mL). Conversely, a low antiproliferative effect was observed in normal CCD18 colon cells (control) compared to tumor cells. Interestingly, the ethanolic extract of *Euphorbia lathyris* also showed a significant antitumor effect against the glioblastoma multiforme cell line A-172 (IC50: 18.58 µg/mL) and was moderately active against other glioblastoma cells (LN-229, SF-268, and SK-N-SH). However, the assay conducted in pancreatic adenocarcinoma (PANC-1) and breast cancer (MCF-7) cell lines showed lower antiproliferative activity (IC50: 185.76 ± 25.8 µg/mL and 89.57 ± 6.29 µg/mL, respectively). This is the first time that an ethanolic extract from the seed of *Euphorbia lathyris* has been tested against glioblastoma multiforme cell lines, pancreatic adenocarcinoma, and breast cancer cell lines. In previous studies, esculetin alone induced apoptosis in the human breast cancer cell line ZR-75-1 via mitochondrial apoptotic pathways [48], as well as in the pancreatic cancer cell lines PANC-1, MIA PaCa-2 and AsPC-1 (IC50 of 100 µM) [49].

Molecular analysis of the mechanisms by which the ethanolic extract induced cell death suggested a double pathway based on apoptosis mediated by caspases and autophagy. Recent studies showed that esculetin alone inhibited proliferation in LoVo colon cancer cells, inducing arrest in the G0/G1 phase and increasing the propapoptotic proteins caspase 3, 7, and 9 [50,51]. Similar results were obtained by Wang et al. [52] in leukemia cell lines, which exhibited apoptosis and autophagy after treatment with esculetin. These results support those observed with the use of our ethanolic extract, which also activated both mechanisms, suggesting that the effect may be associated to a large extent with the presence of esculetin. However, our results clearly demonstrated that the exposure of tumor cells to the extract led to activation of the intrinsic apoptosis pathway, with a significant increase in the expression of cleaved caspases 9 and 3, and the extrinsic apoptosis pathway with an increase in the expression of cleaved caspase 8.

Furthermore, in the present study, effects of the ethanolic extracts from the defatted flour of *Euphorbia lathyris* on angiogenesis, metastatic potential, and CSCs have been described for the first time. Previously, Park et al. [53] detected that cultured HUVECs exposed to esculetin reduced the synthesis of VEGF without causing cytotoxicity. In our study, we did not incubate HUVECs with the ethanolic extract. Instead, we obtained a conditioned medium that contained only tumor cell factors produced by colon cancer cells incubated with the functional extract. When HUVECs were treated with the conditioned medium (extract-free), a significant inhibition of blood vessel formation was observed, suggesting that the extract was able to modify tumor cells influencing HUVECs and simultaneously preventing the formation of blood vessels. These results suggest that the antitumor activity of the extract may include an antiangiogenic effect, which has been widely related to tumor progression [54]. On the other hand, non-cytotoxic doses of the ethanolic extract induced a significant decrease in tumor cell migration, which was more evident at 72 h (18.7%), and a significant decrease in CSC subpopulations. These undifferentiated and pluripotent cells show a high tumorigenic capacity and therapeutic resistance (chemo-resistance), being responsible for clinical recurrence and the failure of current chemotherapeutic regimens [55]. Our results are clinically relevant because not only do many of the current chemotherapeutic regimens not affect CSCs but they also select this subpopulation of cells, promoting early tumor recurrence and increasing its aggressiveness [56].

## 5. Conclusions

Unlike the aggressive extraction methods used in most studies conducted on *Euphorbia lathyris*, we developed a new ethanolic extract from the defatted flour of seeds of *Euphorbia lathyris* (variety S3201) following a simple and inexpensive methodology. This extract showed a high proportion of polyphenols, highlighting the presence of esculetin, euphorbetin, gaultherin, and kaempferol-3-rutinoside. In addition, it exhibited significantly high antitumor activity against colon cancer cells (both resistant and non-resistant) and glioblastoma multiforme cells via the activation of apoptosis (intrinsic and extrinsic pathways) and autophagy. Interestingly, in the T84 cell line, the ethanolic extract also showed decreased colon cancer cell migration, antiangiogenic capacity, and reduction of CSC subpopulations, which are clearly related to tumor relapse and aggressiveness. This novel antitumor agent offers great potential to develop new and more effective therapeutic strategies against colon cancer. Future studies will be necessary to determine its potential applicability and the plausibility of conducting clinical trials.

## 6. Patents

Results have been protected by the patent application Ministerio de Ciencia e Innovación P202030454.

## Figures and Tables

**Table 1:** Analysis of yield and antioxidant activity of the ethanolic extract from the defatted flour of mature seeds of *Euphorbia lathyris*.

Euphorbia Lathyris	Yield *	Total Polyphenols **	Antioxidant Activity **
Ethanolic extract from defatted flour of mature seeds	117.5 ± 9.22	33.5 ± 5.79	23.0 ± 1.92

* (mg/g flour); ** (μg GA equivalents/mg extract). Data represent the mean value ± SD of triplicate.

**Table 2:** Identification of bioactive compounds in the ethanolic extract from the defatted flour of *Euphorbia lathyris*.

Compound	MF	[M-H]-	RT	PPM	% Conf.
Esculetin	C_9_H_6_O_4_	177.0181	2.62	−4	90–100
		177.0185	2.61	−1.7	
Euforbetin	C_18_H_10_O_8_	353.0298	3.17	−0.6	90–100
		353.0301	3.14	1.1	
Gaultherin	C_19_H_26_O_12_	445.1349	1.89	0.7	90–100
		445.1351	1.81	1.1	
Carnosol	C_20_H_26_O_4_	329.1744	6.1	−2.7	90–100
		329.175	6.11	−0.9	
Kaempferol-3-rutinóside	C_27_H_30_O_15_	593.151	3.86	0.7	90–100
		593.152	3.87	2.4	

TR: retention time; MF: molecular formula; PPM: error; MS: mass; % Conf: reliability percentage.

**Table 3:** Quantification of bioactive compounds of the ethanolic extract from the defatted flour of *Euphorbia lathyris*.

	Esculetin	Kaempferol-3-Rutinoside
ppm (mg/L)	267.7 ± 34.2	25.8 ± 3.27
mg compound/100 mg extract	0.41 ± 0.12	0.03 ± 0.002

Standards of esculetin and kaempferol-3-rutinoside were used to the determination. Data represent the mean value ± SD of triplicate.

**Table 4:** Cytotoxicity activity (IC50) of the ethanolic extracts from the defatted flour of *Euphorbia lathyris* in colon cancer and non-tumor colon cell lines.

	IC50 (μg/mL)		
	T84	HCT15	CCD18
Ethanolic extract from defatted flour of mature seeds	16.3 ± 2.54	72.9 ± 1.27	266.0 ± 18.5
5-Fu (µM)	2.68 ± 0.16	6.58 ± 0.35	7.35 ± 0.41

Data are presented as mean ± standard deviation of triplicate analysis.

**Table 5:** Cytotoxicity activity (IC50) of the ethanolic extract from the defatted flour of *Euphorbia lathyris* in human glioblastoma multiforme, pancreatic adenocarcinoma, and breast cancer cell lines.

	IC50 (μg/mL)					
	SF-268	SK-N-SH	A-172	LN-229	PANC-1	MCF-7
Ethanolic extract from defatted flour of mature seeds	39.3 ± 13.2	71.4 ± 13.6	18.6 ± 1.64	70.5 ± 4.48	185.8 ± 25.8	89.6 ± 6.29

Data are presented as mean ± standard deviation of triplicate analysis.

## Data Availability

Not applicable.

## Supplementary Materials

The following are available online at https://www.mdpi.com/2072-6643/13/2/566/s1, Table S1. Primer sequences used to analyze CSCs markers.
